# The Effect of Metformin in Experimentally Induced Animal Models of Epileptic Seizure

**DOI:** 10.1155/2019/6234758

**Published:** 2019-02-04

**Authors:** Ebrahim M. Yimer, Awol Surur, Dawit Zewdu Wondafrash, Abadi Kahsu Gebre

**Affiliations:** Mekelle University, College of Health Sciences, Department of Pharmacology and Toxicology, Ethiopia

## Abstract

**Background:**

Epilepsy is one of the common neurological illnesses which affects millions of individuals globally. Although the majority of epileptic patients have a good response for the currently available antiepileptic drugs (AEDs), about 30-40% of epileptic patients are developing resistance. In addition to low safety profiles of most of existing AEDs, there is no AED available for curative or disease-modifying actions for epilepsy so far.

**Objectives:**

This systematic review is intended to evaluate the effect of metformin in acute and chronic animal models of an epileptic seizure.

**Methods:**

We searched PubMed, SCOPUS, Sciences Direct, and grey literature in order to explore articles published in English from January 2010 to November 2018, using key terms “epilepsy,” “seizure,” “metformin,” “oral hypoglycemic agents,” and “oral antidiabetic drugs”. The qualities of all the included articles were assessed according to the Collaborative Approach to Meta-Analysis and Review of Animal Data from Experimental Studies (CAMARADES).

**Results:**

Out of six hundred fifty original articles retrieved, eleven of them fulfilled the inclusion criteria and were included for final qualitative analysis. In these studies, metformin showed to control seizure attacks by attenuating seizure generation, delaying the onset of epilepsy, reducing hippocampal neuronal loss, and averting cognitive impairments in both acute and chronic models of an epileptic seizure. The possible mechanisms for its antiseizure or antiepileptic action might be due to activation of AMPK, antiapoptotic, antineuroinflammatory, and antioxidant properties, which possibly modify disease progression through affecting epileptogenesis.

**Conclusion:**

This review revealed the benefits of metformin in alleviating symptoms of epileptic seizure and modifying different cellular and molecular changes that affect the natural history of the disease in addition to its good safety profile.

## 1. Introduction

Epilepsy is a highly prevalent neurological condition characterized by an abnormality in electrical excitability of a group of neurons. This disease threatens about 50 million people worldwide, and approximately three fourths of them reside in low-income countries. The disease has neurobiological, cognitive, psychological, and social consequences leading to a substantial morbidity, mortality, and low quality of life [1]. This problem gets worse in developing countries where approximately 90% of epileptic patients are not receiving proper antiepileptic drugs. As a result, people with epilepsy remain to be stigmatized and have a lower quality of life (QoL) compared to people with other chronic medical disorders [2–4].

Various medical conditions including seizure incidence and severity, antiepileptic drug-associated adverse effects, and psychological factors such as depression, anxiety, fear of losing control, worries about seizure occurrence, and negative coping are among the contributing factors for poor outcomes of epilepsy [5].

In spite of the availability of a dozen of antiepileptic drugs (AEDs) with varying mechanisms, the overall outcome and quality of life of epileptic patients have not been improved substantially. This might because of the following reasons: (i) the currently available AEDs provide only a symptomatic relief without influencing the epileptogenesis. (ii) They have low safety profile particularly with the old-generation AEDs [6–10]; hence, the safety issue of existing AEDs is questionable in pregnant and lactating mothers who are receiving more than one AED. (iii) Nearly one third to one half of patients with epilepsy failed to respond to the currently available AED, “drug-resistant epilepsy” [5, 11], of which 70% were patients who have temporal lobe epilepsy. Temporal lobe epilepsy (TLE), in turn, is highly associated with poor quality of life, intensified psychological and physical morbidities, and increased sudden and unexplained mortality [12]. Drug resistance in epilepsy is related to increased rates of death, disability, psychosocial illness, and compromised QoL. Besides, it has significant implications in terms of costs [4]. (iv) Most of the existing AEDs are highly associated with marked adverse effects [13], which can contribute to drug discontinuation because of intolerance to adverse effects. (v) Many of the existing AEDs were also reported to have complex drug-drug interactions [14, 15] that adversely contribute for underdosing (treatment failure) or overdosing of their own or other concomitantly administered medications. Considering these limitations of existing drugs, exploring new antiepileptic drugs having a better efficacy and safety remains important.

Metformin is the first-line antidiabetic agent which is primarily used for the treatment of type II diabetes mellitus because of its efficacy and tolerability [16, 17]. In addition, recent studies have shown its anticancer [18–20], antitubercular [21, 22], antioxidant properties [23–25], and neuroprotective [26] actions. Multifarious molecular and cellular events are thought to involve in the initiation and progression of epileptogenesis. Numerous studies have shown the potential role of metformin to modify these cellular and molecular alterations in an animal model of various neurological disorders [27–31], including improvement in spatial memory, learning, cognition, and neuronal plasticity [32–36] and modulation of proinflammatory cytokines as well as markers of oxidative stress [36–38].

## 2. Methods

### 2.1. Searching Strategies

Articles were retrieved through a systematic search using different electronic databases, including PubMed, SCOPUS, and Sciences Direct as well as manual search from grey literature. The keywords/phrases employed were metformin and oral hypoglycemic and antidiabetic agents in combination with epilepsy and seizure, then search terms were combined using either “AND” or “OR” between two or more terms (Table 1). The searches were restricted to articles published in English language only.

### 2.2. Inclusion and Exclusion Criteria

All studies that are intended to assess the effect of metformin in acute and/or chronic epileptic seizure models and published from January 2010 to November 2018 were included in this systematic review. The search was limited to original articles published in English language only irrespective of the sample size used and/or duration of follow-up. All articles with the intervention of metformin attempt to control chemical-induced seizure in animal epileptic seizure models were included as long as the outcomes were clearly documented. From the included studies, the effects of metformin on seizure frequency, onset, severity, or complete seizures termination were taken as primary outcomes. Conversely, the effects of metformin in chemical-induced and electrical kindling epileptic seizure related to oxidative stress, apoptosis, neuroinflammation, neurogenesis/neurodegeneration, and other markers associated with epileptic seizures were considered as secondary outcomes. However, duplicated articles and studies with incomplete, redundant, and unclearly defined outcome measures were excluded.

### 2.3. Data Extraction and Synthesis

The initial screening of the articles by title, abstract, and full text was carried out by two authors (EMY and AS) independently based on the predefined inclusion and exclusion criteria. After each selection round (title, abstract, and full texts), the authors met and resolved any discrepancy by discussion, while potential disagreements were solved by the involvement of the third author (AKG). The lists of reference of the entire full-text articles were appraised to ascertain additional articles of relevance that were necessary to retrieve the full text. The qualities of all the included studies were substantiated. Finally, all the included accessible full-text article data were extracted to assemble appropriate information on study designs/protocols, interventions given, and main treatment outcomes.

### 2.4. Quality Assessment

**Figure 1:**
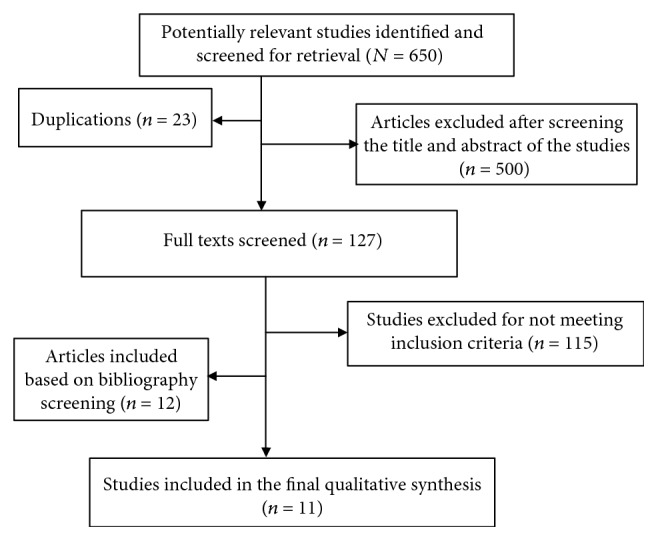
Flow chart of the article screening process for qualitative analysis based on the predefined inclusion and exclusion criteria.

The methodological quality of all the included articles was assessed in accordance to the Collaborative Approach to Meta-Analysis and Review of Animal Data from Experimental Studies (CAMARADES) with slight modification [39] of the 10-item quality checklists as follows: (1) peer-reviewed publication, (2) assignment of experimental and control groups, (3) housing and husbandry conditions, (4) intervention/exposure group procedure, (5) random allocation of animals to the assigned group, (6) concealment of allocation, (7) blind assessment of treatment outcomes, (8) biochemical evaluation, (9) histopathological assessment, and (10) description of statistical analysis (Table 2). Each item was given either one point if it satisfied the criteria or zero if insufficiently described or not explained at all. The two authors have independently assessed the study quality, and the final result was cross-checked and arbitrated by discussion in case of disagreement. Finally, the overall quality score was calculated and expressed as mean ± standard error of the mean. A total of 11 articles were reviewed for this qualitative analysis (Figure 1).

## 3. Results and Discussion

In the present review, we extracted data related to the potential effects of metformin in experimentally induced acute and chronic epileptic seizure of animal models. A total of 650 articles were retrieved from different sources, of which eleven of them were included for the final qualitative analysis (Figure 1).

### 3.1. Study Quality Assessment

The quality appraisal was undertaken for each of the included studies independently according to the Collaborative Approach to Meta-Analysis and Review of Animal Data from Experimental Studies (CAMARADES) before data extraction. According to the CAMARADES quality assessment obtained, all articles included were published in peer-reviewed journal, an intervention/exposure group allocation was clearly stated, and a proper and well-explained statistical analysis was applied. The quality scores for all the included studies were within acceptable range (greater than or equal to 5), but none of the studies reported the allocation concealment (blinded induction of the model and assessment of the outcome). The studies conducted by Mehrabi et al. [40] and Zhao et al. [41] scored the highest score (9/10). However, two studies done by Sánchez-Elexpuru et al. [42] and Bibi et al. [43] have earned the lowest scores (5/10), while the remaining seven studies scored between six and eight. The overall mean quality score of the articles was 7.36 with 0.43 standard error of the mean [5–9] (Table 2).

Animal models of epileptic seizures have played an indispensable role in advancement of our knowledge on the basic mechanisms of epileptogenesis and have been instrumental in the preclinical development of new antiepileptic agents [52]. Among different chemicals used to induce an epileptic seizure in animals, kainic acid and pilocarpine have been the most commonly used agents to induce temporal lobe epilepsy (TLE), status epilepticus, and other epilepsy types and provide a better understanding of the process of epileptogenesis [52–54].

Metformin is a well-known first-line oral antidiabetic agent, but recent studies also showed the benefits of metformin in neurological disorders including epilepsy. Several studies have shown the antiseizure activity of metformin against experimentally induced (chemicals or electrical kindling) acute and chronic animal models of epileptic seizure (Table 3). Evidence from these studies suggested that metformin has a pronounced antiepileptic potential in PTZ-induced acute seizure as well as kainic acid and pilocarpine-induced epileptic seizure models via delaying the onset seizure, reducing seizure frequency and duration, facilitating seizure termination, and attenuating oxidative stress, neuroinflammatory markers, and different proteins which primarily evolved in the initiation or propagation of epileptic seizure. Metformin-treated animals also showed improved behavioral and cognitive performance, reduced PTZ-induced mortality, and suppressed *α*-synuclein expression in the hippocampal CA3 region.

In this review, we have also included the effects of metformin in experimentally induced lafora disease (LD). We included these studies since LD is a rare and progressive myoclonic seizure characterized by focal and generalized seizure, neurological dysfunction, myoclonic and absence seizure, and cognitive decline [42, 55], and more importantly, the outcome measure of these studies was seizure-like activity and neurological and mortality outcomes of metformin treatment, which are the primary interest of this review.

There is also some evidence that pointed out that diabetes mellitus is expressively associated with increased risk of epilepsy compared to nondiabetic patients [56–58]. Hence, it is fascinating that metformin might be a potential and promising pharmacological agent particularly for patients who have concurrent conditions of diabetes mellitus and epilepsy. Owning to this foresight, continual clinical studies are essential to be carried out in order to make sure the efficacy of metformin in epileptic patients as well as in patients who have comorbidities of diabetes and epilepsy.

### 3.2. Possible Mechanisms of Antiseizure/Antiepileptic Actions of Metformin

After induction of seizure either by chemicals or by electrical kindling, it is known that animals exhibit behavioral changes before or after the actual onset of seizure. In the long term, seizures also cause cognitive impairments. However, in these studies metformin treatment reduced behavioral manifestations including touch-response, pick-up, and finger snap and improved cognitive decline. Moreover, metformin reduced seizure severity parameters including mortality, seizure score, and duration of seizure experience while it increased the latency for the first onset of seizure. All these evidences suggested the potential roles of metformin to prevent the symptoms associated with epilepsy.

To date, there is no AED available for curative or disease-modifying actions of epilepsy. Hence, the present therapeutic approach is symptomatic management and supportive care in order to improve the longevity and quality of life of the patients [59, 60]. The epileptogenesis process involves several molecular and cellular changes which can be used as the potential targets for treatment and prevention of epilepsy, though most of the currently available drugs work just by suppression of seizure without affecting the underlying pathological conditions. Metformin was found to prevent some of the cellular changes that underlie the epileptogenesis process including neuronal cell loss, gliosis, and apoptosis which are among the well-known cellular changes observed in epilepsy [61–63]. Metformin is also capable of deterring the molecular alterations including oxidative stress which is a peculiar factor that plays an enormous role in the initiation and progression of epileptogenesis [64, 65].

Mitochondrial dysfunction and abnormal gene expression of oxidative markers involved in scavenging reactive oxygen and nitrogen species have resulted in a profound increment of free radicals and impairment of brain mitochondrial oxygen consumption. All these are suggested to contribute to epileptogenesis [65–69]. Several clinical studies also showed that there is an impairment of biological enzymatic and nonenzymatic antioxidants and overexpression of free radicals in epileptic patients compared to normal control. Most of these epileptic patients who displayed an imbalance oxidative status were refractory to existing AEDs [70–74]. Furthermore, antioxidant agents and targeting oxidative stress were showing a promising antiepileptic potential by attenuating seizure generation, delaying the onset of epilepsy, arresting disease progression, reducing hippocampal neuronal damage, and averting cognitive impairments in different animal models of an epileptic seizure [64, 70, 75]. Interestingly, metformin showed antioxidant activities through attenuation of oxidative free radicals including lipid peroxidation (thiobarbituric acid-reactive substances and malondialdehyde) and advanced glycation end-products and improved the antioxidant defense system including superoxide dismutase, catalase, and glutathione levels [23, 25, 41, 50, 76] in addition to its effects on seizure outcomes.

There is also growing evidence that suggests the activation of inflammatory processes in patients and animal models of epilepsy and the epileptogenic effect of several inflammatory mediators acting on glia and neurons both directly and indirectly to influence neuronal excitability [77–79]. All these evidences are further strengthened by the beneficial effects of nonsteroidal anti-inflammatory drugs and other anti-inflammatory molecules on epileptic and epileptogenic outcomes against animal models of epileptic seizure [77, 80, 81]. Similarly, various studies showed the ability of metformin in mitigating the release and production of endogenous proinflammatory mediators including *Phospho*-*IkBα*, tumor necrosis factor alpha (TNF*α*), interleukin 1 beta (IL-1*β*), IL-6, and vascular endothelial growth factor (VEGF) [82–85].

Selective neuronal loss from the limbic brain area and hippocampal CA1/CA3 subregion is the major neurobiological feature noted in epileptic patients and animal models of epilepsy. In two thirds of patients with TLE, seizures arise from foci in the hippocampus, amygdala, and piriform cortex, which are the areas of extensive neuronal loss [53, 86]. This shows the possible involvement of neurodegeneration in epilepsy. Metformin is reported to prevent neuronal loss via inhibition of microglia activation-mediated inflammation via NF-*κ*B and mitogen-activated protein kinase (MAPK) signaling pathway [87]. The long-term upregulation of the brain-derived neurotrophic factor (BDNF) in the hippocampus showed to enhance epileptogenesis [88, 89], while metformin treatment attenuated the PTZ-induced overexpression of BDNF and its receptor [40]. Metformin also exhibited a substantial reduction of cellular apoptosis induced by PTZ by modifying the expression of caspase-3 and -9 in metformin-pretreated epileptic mice [43]. The observed antiepileptic effect of metformin could be as the result of its effect on all or some of the stated changes. Hence, the therapeutic approach towards molecular apoptosis (either intrinsic or extrinsic pathway) and neuroinflammation can be an emerging area that potentially serves as the neuroprotective role in epilepsy [90, 91].

Impairment in tricarboxylic acid (TCA) cycle turnover was noted in animal models of epilepsy [92, 93]. This suggests an alteration of amino acid and glucose metabolism during the course of epileptogenesis [94]. Alvestad et al. have also shown the reduction of glucose metabolism to glutamate in the hippocampal formation and entorhinal cortex of kainic acid-induced epileptic rats which indicate TCA cycle dysfunction [95]. On the contrary, metformin reduced the glucose level in the animal model of epilepsy which may attribute for better outcomes in epilepsy [45]. This might also be via CtBP mediated as metformin was found to increase CtBP, which is known to have antiglycolytic activity [96]. In addition, metformin reduced the albumin level [45] which is known to contribute to neuronal death observed in epilepsy [97, 98].

The molecular mechanism of metformin behind these effects could be due to its capacity to activate MAPK thereby inhibiting the downstream signaling pathway including mammalian target of rapamycin (mTOR) signaling, PI3K/Akt signaling, neuroinflammation via suppression of MAPK-NF-*κ*B, the release of reactive oxygen species, and production of toxic proteins [46, 48, 99]. Conversely, a recent study conducted by Rubio Osornio et al. showed that metformin administration alone or along with caloric restriction suppressed the expression of the mTOR gene in an electrical kindling TLE mouse model, which might be a compensatory mechanism due to inhibition of the mTOR pathway [51]. Similarly, there are a number of studies that demonstrate the role of metformin in neuroprotection, enhancement of spatial memory, and neurogenesis, which is mediated through the AMPK and atypical protein kinase C and aPKC-CBP pathway in various models of CNS disorders [27, 34, 100–102].

Despite that the neurobiological basis of *α*-synuclein protein aggregation in epilepsy is inconclusive, there is growing of evidence that showed its association with both intractable and newly diagnosed epileptic patients [103, 104]. A study conducted by Hussein et al. showed that metformin treatment significantly downregulated *α*-synuclein expression in a PTZ-induced animal model of epileptic seizure [50], which requires further studies to establish the association of this protein and an epileptic seizure and also the persistent suppression of metformin against *α*-synuclein accumulation.

## 4. Conclusion

Generally, this review revealed that metformin can alleviate symptoms of epileptic seizure in addition to its potential roles to modify the molecular and cellular changes including oxidative stress, neuroinflammation, apoptosis, and neuronal loss observed during the initiation and progression of the disease. The good safety profile coupled with the multiple mechanisms of metformin counteracting epileptic seizure is a promising aspect of the drug in the treatment of epilepsy. However, available evidences so far are preliminary; further preclinical and clinical studies are required in order to determine its long-term efficacy and safety in epilepsy.

## Figures and Tables

**Table 1:** Databases employed and respective key terms used.

Databases and search terms used to extract all available and relevant articles	No. of articles retrieved (*n*)
SCOPUS	*n* = 354
1 “seizure” OR “epilepsy” AND “metformin”	
2 “seizure” OR “epilepsy” AND “oral hypoglycemic agents” OR “oral antidiabetic agents”	
Sciences-Direct	*n* = 237
1 “seizure” OR “epilepsy” AND “metformin”	
2 “seizure” OR “epilepsy” AND “oral hypoglycemic agents” OR “oral antidiabetic agents”	
PubMed	*n* = 28
1 “epilepsy [Mesh] OR epilepsy^∗^[tw]”	
2 “seizure [Mesh] OR seizure^∗^[tw]”	
3 “metformin [Mesh] OR Metformin^∗^[tw]”	
4 “oral hypoglycemic agents [Mesh] OR Oral hypoglycemic agents^∗^[tw]”	
Finally, search for (#1 OR # 2) AND (# 3 OR # 4)	
Grey literatures (Google scholar and regular Google search)	
(1) Antiepileptic activities of metformin OR epilepsy and metformin	
(2) Antiseizure actions of metformin OR seizure and metformin	

**Table 2:** The quality assessment of individual study obtained according to the CAMARADES checklist items [39, 44].

References	Criteria										
	I	II	III	IV	V	VI	VII	VIII	IX	X	Total (of 10)
Mehrabi et al. [40]	1	1	1	1	1	0	1	1	1	1	9
Zhao et al. [41]	1	1	1	1	1	0	1	1	1	1	9
Bibi et al. [43]	1	1	0	1	0	0	0	1	0	1	5
Sánchez-Elexpuru et al. [42]	1	1	1	1	0	0	0	0	0	1	5
Azeez [45]	1	1	1	1	1	0	1	1	0	1	8
Yang et al. [46]	1	1	1	1	1	0	1	1	0	1	8
Stone et al. [47]	1	1	0	1	1	0	1	0	0	1	6
Berthier et al. [48]	1	1	1	1	1	0	0	1	1	1	8
Chen et al. [49]	1	1	0	1	1	0	1	1	1	1	8
Hussein et al. [50]	1	1	1	1	1	0	1	1	1	1	8
Rubio Osornio et al. [51]	1	1	0	1	0	0	1	1	1	1	7
									Mean quality scores		7.36 ± 0.43 (5 to 9)

(I) Publication in peer-reviewed journal, (II) number of experimental and control groups, (III) housing and husbandry conditions, (IV) details of intervention/exposure group procedures, (V) random allocation to groups, (VI) concealment of allocation, (VII) blinded assessment of outcome**s**, (VIII) biochemical evaluations, (IX) histopathological evaluations, and (X) statistical analysis. 1: criterion is satisfied, 0: the criterion is insufficiently described or not explained at all; the mean quality score is expressed as mean ± standard error of the mean (SEM) and (minimum and maximum score).

**Table 3:** The effects of metformin against epileptic seizure on different animal models.

Model	Methods and intervention	Main treatment outcomes	References
Chronic phase of pilocarpine-induced seizures in model of TLE (Wistar rats)	After induction of seizure, animals were randomly divided into the following: (a) the control group received vehicle, (b) the epileptic group received 360 mg/kg IP pilocarpine, and (c) the treatment group received IP injections of metformin (250 mg/kg/day).	7th day post-treatment, metformin Counteract touch-response, pick-up, and finger snap Decreased BDNF and Trk expression significantly Increased expression of CtBP after drug administration Increased the protein expression of p-AMPK Decreased the protein expression of p-mTOR	Mehrabi et al. [40]
Oxidative damage and PTZ-induced kindling (adult male mice)	Animals were randomly divided into (a) control group, (b) PTZ group, (c) the PTZ + MET group that received MET in dose of 200 mg/kg, and (d) the MET group that received 200 mg/kg of metformin alone.	Pretreatment with metformin: Significantly suppressed the progression of kindling as evidenced by the decrease in seizure scores. Improved the cognitive performance. A noticeable decrement in the concentration of malondialdehyde (MDA). There is also substantial upregulation of glutathione (GSH) level	Zhao et al. [41]
PTZ-induced apoptotic neurodegeneration in human cortical neuronal cells.	HCN-2 cell line derived from the brain tissue of patients having intractable seizures. HCN-2 cortical cells cultured and further divided into (a) control group, (b) PTZ (30 mM) group, and (c) metformin (20 mM) + PTZ group.	Metformin notably reversed the effect of neuronal cell loss compared to the control group. It also prevented PTZ-induced apoptotic neuronal loss by decreasing the expression of caspase-3 and 9. Besides, metformin showed its protective effect by reversing the effect of PTZ-induced neurodegeneration.	Bibi et al. [43]
PTZ-induced seizures in a malin knockout (KO) model of Lafora disease (male mice)	Four groups of 16 adult male mice were analyzed per condition: (a) wild-type mice, (b) malin knockout mice, (c) malin knockout mice with 4-PBA treatment, and (d) malin knockout mice with metformin treatment.	Metformin treatments decreased the overall number (%) of mice developing seizures. Decreasing this percentage below wild-type levels after metformin treatment and PTZ-induced mortality also decreased to 0%. Metformin treatments increased the latency for PTZ-induced seizure onset and shorten seizure lengths in malin KO mice Metformin treatment also attenuated the hyperexcitability detected in mice lacking the malin protein.	Sánchez-Elexpuru et al. [42]
Biochemical parameters in PTZ- induced epileptic rats	Rats were divided into 3 groups: (a) group 1 served as control, (b) group 2 were treated with glibenclamide at a dose of 5 mg/kg, and (c) group 3 were treated with metformin at a dose of 150 mg/kg.	Only rats treated with metformin showed a decrease in serum glucose level after 3 and 24 hours, increasing after a week and returning near normal. Metformin treated group showed a significant increment of serum TC level after 3, 24 hours, without effect after a week. Metformin also displayed a substantial upsurge of serum TP level after 3 hours. Further, the metformin-receiving group showed a marked reduction in serum albumin and globulin levels	Azeez [45]
Kainic acid and PTZ-induced seizures (adult male mice)	The acute seizures were induced by IP injection of PTZ (70 mg/kg) while the chronic seizure model was established by kainic acid.	Mice that received metformin treatment for 30 days Behavioral assay showed that the Racine score was not significantly different between metformin-treated and control groups. The incidence of GTCS was also not different in the metformin group (8/12) and control group (9/12) (*P* > 0.05). But mice treated with metformin displayed a substantial reduction of mortality (3/8) compared to the control group (8/9). Metformin-treated mice showed a significantly increment in p-AMPK level in the PTZ-induced acute seizure model. Chronic metformin treatment facilitates seizure termination in PTZ-induced acute seizures and also promotes termination of chronic seizures (by reducing the duration of SLE). Long-term metformin treatment also showed considerable upregulation of the level of p-AMPK.	Yang et al. [46]
Yeast/cornmeal/agar media- induced seizure in *D. melanogaster*	Two-day old seizure-sensitive flies were fed (1 g standard yeast/agar media or 1 g of media + 25 mg of metformin) for 2 days. The movement or SLA using the HandyAvi software program was recorded.	The seizure-sensitive flies in the metformin-receiving group showed a reduction in the SLA path length expressively as compared to control. The SLA duration was also significantly reduced whereas SLA velocity was not changed considerably.	Stone et al. [47]
Lafora disease (LD) model in LD hybridized mouse	After hybridization, heterozygous mice of *Epm2b+/-* (used as control) and homozygous *Epm2b-/-* (malin KO) were used. 2% trehalose and 20 mM of 4-PBA as positive control and 12 mM of metformin were administered as a test substance.	After 2 months of treatment The metformin-receiving group displayed an enhanced activation of AMPK in both control and KO malin mice. But metformin-treated animals produced an insignificant rise in the level of chaperone BiP/GRP78. Treatment with metformin also showed a noticeable decrement of the number of PAS^+^ aggregates. Both 4-PBA and metformin treatments prevented the neuronal loss and hippocampal gliosis compared to the trehalose-treated group. 4-PBA or metformin also ameliorates some *Epm2b*-/- neuropsychiatric symptoms.	Berthier et al. [48]
PTZ- induced SE model in Sprague-Dawley rats	The rats were randomly divided into (a) control (saline), (b) SE (PTZ), (c) SE + salubrinal, (d) SE + GSK2656157 (GSK), (e) SE + metformin (200 mg/kg); (*n* = 12/group).	The metformin-receiving group showed a marked reduction in CHOP expression (*p* ≤ 0.002) compared to the SE group. Even if a reducing tendency was observed at 24 hours following metformin treatment, CHOP expressions were not significantly different in all of the treatment groups between 6 and 24 hours. On top of CHOP, eIF2*α* and PERK levels were also reduced in the metformin-treated group compared to SE control. The rate of apoptosis was meaningfully reduced in the metformin-receiving group as compared to the SE group.	Chen et al. [49]
PTZ-induced epilepsy model in Sprague-Dawley rats	Animals were randomly allocated into (*n* = 10 rats/group) (i) normal group: received saline, (ii) metformin group like normal group but received metformin (200 mg/kg) pretreatment daily for two weeks, (iii) PTZ group: rats received PTZ (50 mg/kg) on alternate days for two weeks, and (iv) metformin + PTZ group: like PTZ group but received metformin (200 mg/kg) pretreatment daily for two weeks.	The metformin-treated PTZ group showed meaningful decrement of seizure scores compared to the PTZ group. The metformin-treated PTZ group also displayed noticeably longer values of the seizure onset latency than the PTZ group in almost all trials. Compared to the first trial, the values of the seizure duration in all other trials showed a gradual significant increase in PTZ-administered groups. The metformin-receiving animals of PTZ groups exhibited substantial reduction in seizure duration compared to the PTZ group in all recorded trials. The metformin-treated PTZ group presented a noteworthy reduction of MDA and substantial upregulation of GSH level in the CA3 region of the hippocampus compared to the PTZ group. Conversely, metformin treatment failed to show a significant increase in catalase level as compared to the PTZ group. The metformin-receiving PTZ group displayed meaningful downregulation of apoptotic protein, caspase-3, and *β*-catenin in the hippocampal area compared to PTZ groups. In histopathological analysis, the metformin-treated group also showed a normal shape and number of neurons as well as a substantial reduction of abnormal neurons in the brain tissues. Compared to the PTZ group, the metformin-treated group exhibited a meaningful downregulation of *α*-synuclein expression in the CA3 region of the hippocampus.	Hussein et al. [50]
Electrical kindling of the amygdala; model of TLE in Wistar rats	Animals were grouped into the following: fed ad libitum (AL; *n* = 10), fed ad libitum plus metformin (AM; *n* = 8), and rats subjected to 15% caloric restriction (CR) plus metformin (CM; *n* = 7); metformin administered at a dose of 100 mg/kg daily for 5 days/week till the end of the experiment.	The metformin plus CR- (CM-) treated group showed a significant increment of the after-discharge (AD) threshold, while its length was reduced, hence diminishing the total cumulative AD duration than the AM and AL group. CM also reduced the time spent in seizure stage 5. Consequently, the time spent in generalized convulsive seizures was reduced (stages 4-5). Metformin alone (AM) substantially upregulated AMPK phosphorylation in the neocortex, while CM amplified the phosphorylation of AMPK both in the neocortex and in the hippocampus. AM and CM administration also augmented the expression of the mTOR gene, but reduced PKB phosphorylation in the neocortex and the hippocampus.	Rubio Osornio et al. [51]

SE = status epilepticus; TC: total cholesterol; TP: total protein; SLE: seizure-like events; SLA = seizure-like activity; AMPK: adenosine monophosphate-activated protein kinase; p-AMPK: phosphorylated AMPK; PTZ: pentylenetetrazole; IP: intraperitoneally; TLE: temporal lobe epilepsy; MDA: malondialdehyde; GSH: glutathione; D. melanogaster = Drosophila melanogaster; 4-PBA = 4-phenylbutyric acid; KO = knockout; PAS^+^ = periodic acid-Schiff (+ indicates the presence of polyglucosan inclusions or LB); CHOP = C/EBP homologous protein; eIF2*α* = eukaryotic initiation factor 2*α*; PERK = protein kinase RNA-like endoplasmic reticulum kinase; CR = caloric restriction; mTOR = mammalian target of rapamycin; AL = ad libitum; AM = ad libitum plus metformin; CM = caloric restriction plus metformin.

## Abbreviations

AEDs: Antiepileptic drugs
AMPK: Adenosine monophosphate-activated protein kinase
CAMARADES: Collaborative approach to meta-analysis and review of animal data from experimental studies
GSH: Glutathione
LD: Lafora disease
MDA: Malondialdehyde
mTOR: Mechanistic target of rapamycin
PTZ: Pentylenetetrazole
QoL: Quality of life
SLE: Seizure-like activity
SLE: Seizure-like events
TLE: Temporal lobe epilepsy
BDNF: Brain-derived neurotrophic factor
MAPK: Mitogen-activated protein kinase.
